# Development and validation of a prognostic model incorporating [^18^F]FDG PET/CT radiomics for patients with minor salivary gland carcinoma

**DOI:** 10.1186/s13550-020-00631-3

**Published:** 2020-07-06

**Authors:** Nai-Ming Cheng, Cheng-En Hsieh, Yu-Hua Dean Fang, Chun-Ta Liao, Shu-Hang Ng, Hung-Ming Wang, Wen-Chi Chou, Chien-Yu Lin, Tzu-Chen Yen

**Affiliations:** 1 Department of Nuclear Medicine and Molecular Imaging Center, Chang Gung Memorial Hospital, Chang Gung University College of Medicine, Taoyuan, Taiwan; 2 Department of Nuclear Medicine, Chang Gung Memorial Hospital, Keelung, Taiwan; 3 Department of Radiation Oncology, Chang Gung Memorial Hospital, Chang Gung University College of Medicine, Taoyuan, Taiwan; 4 Department of Radiology, University of Alabama at Birmingham, Birmingham, Alabama USA; 5 Department of Otolaryngology − Head & Neck Surgery, Chang Gung Memorial Hospital, Chang Gung University College of Medicine, Taoyuan, Taiwan; 6 Department of Diagnostic Radiology, Chang Gung Memorial Hospital, Chang Gung University College of Medicine, Taoyuan, Taiwan; 7 Division of Hematology/Oncology, Department of Internal Medicine, Chang Gung Memorial Hospital, Chang Gung University College of Medicine, Taoyuan, Taiwan; 8 Department of Nuclear Medicine, Xiamen Chang Gung Hospital, Xiamen, China

**Keywords:** Minor salivary gland carcinoma, [^18^F]FDG PET/CT, Heterogeneity, Texture analysis, Prognosis

## Abstract

**Objectives:**

The aim of this study was to develop and validate a prognostic model incorporating [^18^F]FDG PET/CT radiomics for patients of minor salivary gland carcinoma (MSGC).

**Methods:**

We retrospectively reviewed the pretreatment [^18^F]FDG PET/CT images of 75 MSGC patients treated with curative intent. Using a 1.5:1 ratio, the patients were randomly divided into a training and validation group. The main outcome measurements were overall survival (OS) and relapse-free survival (RFS). All of the patients were followed up for at least 30 months or until death. Following segmentation of tumors and lymph nodes on PET images, radiomic features were extracted. The prognostic significance of PET radiomics and clinical parameters in the training group was examined using receiver operating characteristic curve analysis. Variables showing a significant impact on OS and RFS were entered into multivariable Cox regression models. Recursive partitioning analysis was subsequently implemented to devise a prognostic index, whose performance was examined in the validation group. Finally, the performance of the index was compared with clinical variables in the entire cohort and nomograms for surgically treated cases.

**Results:**

The training and validation groups consisted of 45 and 30 patients, respectively. The median follow-up time in the entire cohort was 59.5 months. Eighteen relapse, 19 dead, and thirteen relapse, eight dead events were found in the training and validation cohorts, respectively. In the training group, two factors were identified as independently associated with poor OS, i.e., (1) tumors with both high maximum standardized uptake value (SUV_max_) and discretized intensity entropy and (2) poor performance status or N2c-N3 stage. A prognostic model based on the above factors was devised and showed significant higher concordance index (C-index) for OS than those of AJCC stage and high-risk histology (C-index: 0.83 vs. 0.65, *P* = 0.005; 0.83 vs. 0.54, *P* < 0.001, respectively). This index also demonstrated superior performance than nomogram for OS (C-index: 0.88 vs. 0.70, *P* = 0.017) and that for RFS (C-index: 0.87 vs. 0.72, *P* = 0.004).

**Conclusions:**

We devised a novel prognostic model that incorporates [^18^F]FDG PET/CT radiomics and may help refine outcome prediction in patients with MSGC.

## Introduction

Minor salivary gland carcinoma (MSGC)—with an annual incidence of 0.16–0.4 new cases per 100,000 population [1]—is a rare malignancy that accounts for only 0.3–1.8% of all head and neck tumors [2, 3]. The clinical characteristics and histology of MSGC can vary greatly, and the disease course is still largely unpredictable [4, 5]. Several efforts have been made to improve the prognostic stratification of patients with MSGC [4, 6–11]. The American Joint Committee on Cancer (AJCC) tumor stage and the histology risk group according to the 2005 World Health Organization (WHO) classification system [12] are currently considered as the main prognostic factors for both patient survival and disease relapse [11]. Ali S et al. had proposed postoperative nomograms for prediction of major salivary gland malignancy. Five variables were used for predictive for OS: age, clinical T4 stage, histological grade, perineural invasion, and tumor dimension [13]. Lu CH et al. also demonstrated a nomogram including clinicopathologic variables of smoking, tumor grade, perineural invasion, lymphatic invasion, and pathologic T- and N-stage to predict the recurrent probability of major gland cancer [14]. However, surgical and pathological results were required for those nomograms and their utility in MSGC remains unclear.

Growing evidence indicates that [^18^F]FDG parameters can predict outcomes in salivary gland cancer (SGC) [15–18]. We had found in high-risk histology SGC, including 49 MSGC, maximum standardized uptake value (SUV_max_) and discretized intensity entropy (an index of image heterogeneity) having prognostic value [17]. However, their roles in MSGC needed further investigation. In this extended study, we added 26 new cases of MSGC including all kinds of histology, ranging from low to high risk. Owing to major differences in image acquisition, analysis protocols, and different resolution of varied PET systems, PET radiomics studies should meet rigorous methodological and reporting criteria [19–21]—which unfortunately remains a frequently overlooked issue in the available literature (especially in terms of validation and reproducibility).

Starting from these premises, we designed the extended study to (1) develop and validate a prognostic model incorporating [^18^F]FDG PET/CT radiomics for patients with MSGC, (2) compare this model with clinical variables, including staging systems, WHO risk histology types, and previously published nomograms.

## Materials and methods

### Study patients

The inclusion criteria of this study were (1) MSGC patients who complete curative intent therapy between January 2007 and December 2016 at our institutes. (2) All patients must have staging [^18^F]FDG PET/CT before treatment and must follow up of at least 30 months after diagnosis or until death. The exclusion criteria were (1) patients who presented with distant metastases at diagnosis (M1), and (2) patients had a positive history of other malignancies. Demographic and survival data were collected from all participants. Disease staging was performed according to the AJCC Staging Manual, seventh edition [22], based on the results of PET/CT and MRI (or contrast-enhanced CT). In the presence of discrepant findings between the two imaging modalities, lesions were subjected to biopsy. PET images were independently interpreted by two expert nuclear medicine physicians (N.M.C. and T.C.Y.). In accordance with the 2005 WHO classification, the following tumors were considered as high-risk histology types of MSGC: adenoid cystic carcinoma (ACC), mucinous adenocarcinoma, squamous cell carcinoma, small or large cell carcinoma, lymphoepithelial carcinoma, metastasizing pleomorphic adenoma, and high-grade (HG) carcinomas [i.e., HG mucoepidermoid carcinoma (MEC), HG salivary duct carcinoma, HG carcinoma ex-pleomorphic adenoma, and HG cystadenocarcinoma]. Performance status (PS) was assessed using the Eastern Cooperative Oncology Group (ECOG) criteria. Radical surgery was the mainstay of treatment, whereas postoperative radiotherapy (RT) or chemoradiotherapy (CCRT) was performed in selected cases [16]. Patients were treated with definitive RT or CCRT in the presence of at least one of the following conditions: (1) the presence of a non-resectable malignancy; 2) medical intolerance to anesthesia, or 3) unwillingness to undergo surgery. Follow-up imaging consisted of [^18^F]FDG PET/CT and CT or MRI scans performed every 3 − 6 months in the first 2 years, every 6 − 12 months between the third and the fifth year, and every 12 − 24 months thereafter. Using a 1.5:1 ratio, the study sample was randomly divided into a training (*n* = 45) and validation (*n* = 30) group. Randomization was based on the ranking of hospital identification numbers, which were randomly assigned to each patient during their first hospital visit. The optimal cutoff values for PET parameters were determined in the training group and subsequently tested in the validation group. The study followed the tenets of the Helsinki Declaration and was approved by the Institutional Review Board of the Chang Gung Memorial Hospital. Owing to the retrospective nature of the study, the need for informed consent was waived.

### [^18^F]FDG PET/CT image acquisition

Patients underwent pretreatment [^18^F]FDG PET/CT staging within a median of 10 days (range: 1–70 days) from histological diagnosis. Images were acquired in 6-h fasted participants 60 min after the intravenous injection of 370–555 MBq [^18^F]FDG (depending on the patient’s body weight). Forty-eight (64%) and 27 (36%) patients underwent [^18^F]FDG PET/CT on a Discovery ST 16 scanner (GE Healthcare, Milwaukee, WI, USA) and a Biograph mCT scanner (Siemens Medical Solutions, Malvern, PA, USA), respectively. An ordered-subset expectation maximization iterative reconstruction algorithm (4 iterations and 10 subsets for the Discovery ST16, 2 iterations, and 21 subsets for the Biograph mCT) based on CT-based attenuation map were applied for PET image reconstruction. Time-on-flight (TOF) technique to improve image quality was used in the Biograph mCT scanner. The values of axial spatial resolution for the Discovery ST16 and Biograph mCT scanners were 4.80 mm and 2.16 mm, respectively.

### [^18^F]FDG PET radiomics

A fixed 40% threshold (T40) of SUV_max_ was applied for the segmentation of tumors and lymph nodes. This approach has been previously utilized for SGC by other research groups [15] and repeatedly applied in recent studies [23, 24]. Image features were extracted in the volume of interest (VOI) using intensity histogram, gray level co-occurrence matrix (GLCM), gray-level run-length matrix (GLRLM), and gray-level size zone matrix (GLSZM). Intensity histogram is generated using the three-dimensional (3D) tumor volume by discretizing the original intensity into intensity bins. GLCM was used to assess the relationship between two neighboring voxels within the original image in an orientation invariant manner by averaging 13 direction vectors within the neighborhood of one Chebyshev distance. GLRLM determines the size of the uniform run (length) for each gray level. Similar to GLCM, we used the 3D matrix and the values of each texture index are averaged over the 13 directions. GLSZM counts the number of linked voxels which defined as two or more of the neighboring voxels have the same gray level within one Chebyshev distance. To reduce noise stemming from image processing, we applied a fixed bin number method [25] with 16 bins according to our previous paper [17].

SUV_max_ and metabolically active tumor volume (MATV) have prognostic significance in SGC [15, 16] and were therefore included in the analysis. According to PET studies based on double baseline images [25, 26], the following parameters are characterized by high reproducibility and repeatability: asphericity (from shape analysis); discretized intensity entropy (from intensity histogram); angular second moment (ASM) and sum entropy (from GLCM); run-length nonuniformity (RLNU) and high gray level run emphasis (HGLRE) (from GLRLM); zone-size nonuniformity (ZSNU), and high gray level zone emphasis (HGLZE) (from GLSZM). The discretized intensity entropy represents the sum of fixed bins probabilities multiplied by the natural logarithm of the probability values. GLCM features rely on the probability distribution for the elements of the GLCM. ASM measures the feature of textural uniformity of an image and sum entropy quantifies the randomness of intensity distribution. ASM and sum entropy often inversely correlated with each other. RLNU quantifies the distribution of runs over the run lengths, and low RLNU is noted in an image with equally distributed runs along run lengths. HGLRE weights the runs with high gray level voxel intensity. HGLRE is high in an image with greater runs of high intensity. ZSNU measures the distribution of zone counts over the different zone sizes and is expected to be small if the zones are similar throughout the entire image. HGLZE weights the zones with high gray level voxel intensity. HGLZE is high in an image with numerous zones of high intensity.

Because nodal lesions were generally small-sized, we did not apply texture analyses to lymph nodes and only nodal SUV_max_, MATV, and asphericity were taken into account. For patients who had multiple lymph nodes metastases, lymph node MATV was the sum of the volume of all involved nodes while lymph node SUV_max_ and asphericity were determined using the highest value among lesions.

The Chang-Gung Image Texture Analysis toolbox (CGITA) was used for calculation PET parameters. The terms and equations of PET texture parameters and the calculation process are consistent with the Imaging Biomarker Standardization Initiative (IBSI) framework [21, 27]. The compliance of IBSI framework was evaluated using the IBSI digital phantom, and the resulting data were shown in the Supplementary file.

### Statistical analysis

Overall survival (OS) and relapse-free survival (RFS) served as the main outcomes of interest. OS was the time elapsed from the histological diagnosis to the date of death from any cause (or censored on the date of the last follow-up). RFS was defined as the time from the date of primary treatment to the first disease recurrence (or censored on the date of the last follow-up). For patients who did not achieve a complete response after therapy, the date of recurrence was set at the end of primary therapy [28]. Correlations among the study variables were tested using Spearman’s correlation coefficient (ρ). Categorical variables were analyzed using the chi-square test. The difference of PET parameters between training/validation cohorts and scanners was accessed using Mann-Whitney *U* test. To account for a potential selection bias caused by lack of randomization, propensity scores for OS in the training, and validation groups were calculated for different confounding factors. Data analyses were conducted using the SPSS statistical package, version 21 (IBM, Armonk, NY, USA). Statistical testing was two-sided, and Bonferroni’s correction was applied to adjust for multiple comparisons.

The selection of PET parameters for survival analyses was based on three steps. Firstly, the impacts of PET radiomics on OS were assessed in the training group using receiver operating characteristic (ROC) curve analysis. All variables that produced an area under the ROC curve (C-index) significantly different from 0.5 were selected and their optimal cutoff values for OS were determined by method of maximizing Youden index using MedCalc version 19.1 (Mariakerke, Belgium). We set *P* values below 0.10 as statistically significant for the selection process. That was a *P* value of less than 0.01 (0.10/10) was required to declare significance after Bonferroni’s correction. Secondly, subgroup analysis was performed to investigate the interaction and impacts of those parameters on survivals. Finally, clinical factors and potential PET parameters were subsequently entered into multivariate Cox proportional hazards models. The following variables served as covariates in the training group: age, tumor histology, treatment modality, performance status, and clinical AJCC stages. The proportional hazards assumption for each variable was examined by Schoenfeld residuals test. To minimize overfitting of predictor effects during model development thematic series: advanced image analysis and follow the rule of thumb of multivariate analysis [29], multivariate Cox regression model was analyzed using a bootstrap resampling procedure (1,000 samples).

Recursive partitioning analysis (RPA) was applied to devise a prognostic model based on significant clinical parameters and PET radiomics. To this aim, a classification and regression tree (CART) algorithm was applied, and patients were divided according to independent dichotomous variables. To overcome the potential issue of overfitting, fivefold cross-validations (following a random exclusion of 20% of patients in the training group) were repeatedly used for assessing model accuracy. Finally, the resulting survival model was subsequently applied in the validation group using Kaplan-Meier estimates (log-rank test). The predictive ability of different prognostic variables was then compared using the concordance index (C-index) with AJCC staging, WHO high-risk histology for the entire cohort and clinical nomograms for surgical-treated patients. Comparison of C-index was conducted by using a nonparametric approach [30] implanted in MedCalc version 19.1 (Mariakerke, Belgium).

## Results

### Patients and treatment outcomes

The general characteristics of the study patients (*n* = 75) are summarized in Table 1. The median follow-up time in the entire cohort was 59.5 months (range: 2.6–140.9 months) and 76.0 months (range: 31.4–140.9 months) in surviving patients. There was no difference in the follow-up time for training and validation cohorts (median: 55.17 months, range: 2.6–140.9 months vs. 74.6 months, range 7.4–127.9 months, *P* = 0.304). The most common primary tumor site was the oral cavity (*n =* 22, 29.3%), followed by the oropharynx (*n =* 21, 28.0%), nasal cavity/paranasal sinus (*n =* 17, 22.7%), nasopharynx (*n =* 8, 10.7%), hypopharynx (*n =* 3, 4.0%), larynx (*n = 3*, 4.0%), and ear (*n =* 1, 1.3%). The histological diagnoses were as follows: ACC (*n =* 35, 46.7%), MEC (*n* = 25, 33.3%), adenocarcinoma (*n* = 14, 18.7%), and acinic cell carcinoma (*n* = 1, 1.3%). Sixty patients (80.0%) were considered to have tumors with high-risk histology according to the WHO classification. Surgery was the primary treatment modality in 45 study participants (60.0%). The median RT doses delivered to patients who received surgical and non-surgical treatment were 66 Gy (*n* = 38, range: 54 − 76 Gy, 97.4% of cases ≥ 60 Gy) and 72 Gy (*n* = 30, range: 66 − 80 Gy, all cases ≥ 60 Gy), respectively. The hospital identification number did not show any significant association with either T-, N-, and AJCC stages or survival endpoints (OS and RFS). In this extended study, we enrolled MSGC including all kinds of histology and more favorable overall survival (OS) was noted as compared with our previous paper that included only high-risk histology cases (5-year OS: 68.8% vs. 60.4%). Twenty-seven patients (36.0%) died during the study period, and the causes of death were as follows: MSGC, *n* = 22; severe infection, *n* = 1; hypopharyngeal cancer, *n* = 1; pancreatic cancer, *n* = 1; cerebrovascular disease, *n* = 1; and traffic accident, *n* = 1. Disease recurrences were observed in 31 patients (41.3%), with 13, 11, and 7 cases showing locoregional, distant, and concomitant locoregional plus distant recurrences, respectively. No difference in cancer death was noted in the training and validation groups (15 (33.3%) vs. 7 (23.3%) events, Fisher’s exact test, *P* = 0.441). Nineteen (42.2%) and eight (26.7%) patients were dead in the training and validation cohorts, respectively (Fisher’s exact test, *P* = 0.222), with eighteen (41.3%) and thirteen (40.0%) recurrent events noted in the corresponding groups (*P* = 0.814). The training and validation groups did not differ in terms of clinical parameters and there were no differences in propensity scores (median: 0.32 versus 0.29, respectively, *P* = 0.540).

**Table 1:** General characteristics of the study patients

Variable		Entire cohort	Training group	Validation group	*P*
		*n* = 75	*n* = 45 (60.0%)	*n* = 30 (40.0%)	
Age: median [range], years		52 [20-81]	53 [22-78]	51 [20-81]	0.240
Sex	Female	40 (53.3)	25 (55.6)	15 (50.0)	0.646
	Male	35 (46.7)	20 (44.4)	15 (50.0)	
Smoking	Yes	21 (28.0)	12 (26.7)	9 (30.0)	0.797
	No	54 (72.0)	33 (73.3)	21 (70.0)	
Performance	ECOG 0 − 1	72 (96.0)	43 (95.6)	29 (96.7)	1.000
	ECOG 2	3 (4.0)	2 (4.4)	1 (3.3)	
Treatment	Surgery	45 (60.0)	30 (66.7)	15 (50.0)	0.160
	Non-surgery	30 (40.0)	15 (33.3)	15 (50.0)	
WHO histology	High-risk	60 (80.0)	38 (84.4)	22 (73.3)	0.255
	Low-risk	15 (20.0)	7 (15.6)	8 (26.7)	
T-stage	T1 − T2	31 (41.3)	16 (35.6)	15 (50.0)	0.239
	T3 − T4	44 (58.7)	29 (64.4)	15 (50.0)	
N-stage	N0 − N2b	71 (94.7)	42 (93.3)	29 (96.7)	0.646
	N2c − N3	4 (5.3)	3 (6.7)	1 (3.7)	
AJCC stage	I − II	23 (30.7)	12 (26.7)	11 (36.7)	0.445
	III − IV	52 (69.3)	33 (73.3)	19 (63.3)	
Dead events		27 (36.0)	19 (42.2)	8 (26.7)	0.222
Relapse events		31 (41.3)	18 (40.0)	13 (43.3)	0.814

The median time to disease progression after treatment was 15.1 months (range: 2.4–69.1 months). Kaplan-Meier analysis revealed that patients with advanced T-stages, AJCC stages, or who were smokers had worse OS and RFS. Surgery and WHO high-risk histology were found to have an adverse impact on RFS, but not on OS. Patients with ACC tended to have a better OS (*P* = 0.077) but a similar RFS (*P* = 0.957) compared with other histology types. Among patients who were treated with surgery, positive margins, perineural invasion, and lymphatic invasion were identified in 24 (53.3%), 17 (37.8%), and 1 (2.2%) cases, respectively. The median nomogram score for OS [13] was 132 (range: 0-254). The median nomogram score for RFS [14] was 2.76 (range: 1.0-4.0). Notably, patients with advanced N-stage (N2c/N3, *n* = 2/2) or poor PS (ECOG 2, *n* = 3) had 5-year OS and RFS rates of 0%.

### [^18^F]FDG PET radiomics and prognosis

There were significant correlations of MATV with almost every radiomic index, including the following: asphericity, discretized intensity entropy, ASM, sum entropy, RLNU, HGLRE, ZSNU, and HGLZE. Supplementary Table 1 depicts the distribution of all PET parameters. The tumor voxels ranged from 22 to 11412 voxels (median: 245, interquartile range: 129 to 1676 voxels). Three cases (4%) had tumor voxels fewer than 64. Tumor SUV_max_, MATV, asphericity, discretized intensity entropy, ASM, sum entropy, and HGLRE were unaffected by the PET systems. Interestingly, we found that tumor volumes in patients with adenoid cystic carcinoma (ACC, *n* = 35) were larger than other histological types (median MATV: 15.30, range: 1.45-91.30 vs. median: 8.59, range: 1.44-63.18, Mann-Whitney *U* test, *P* = 0.004). But for ACC tumors, more homogeneous uptakes were noted (i.e., significantly lower values of discretized intensity entropy 2.44 (range: 2.05-2.70) vs. 2.53 (range: 2.20-3.25), *P* = 0.011). Twenty-two cases had lymph node disease. PET identified 19 cases. Three cases had small nodal lesions and were noted only in the pathological report after surgery. No different lymph node SUV_max_, asphericity, and MATV was noted, Supplementary Table 1.

**Fig. 1:**
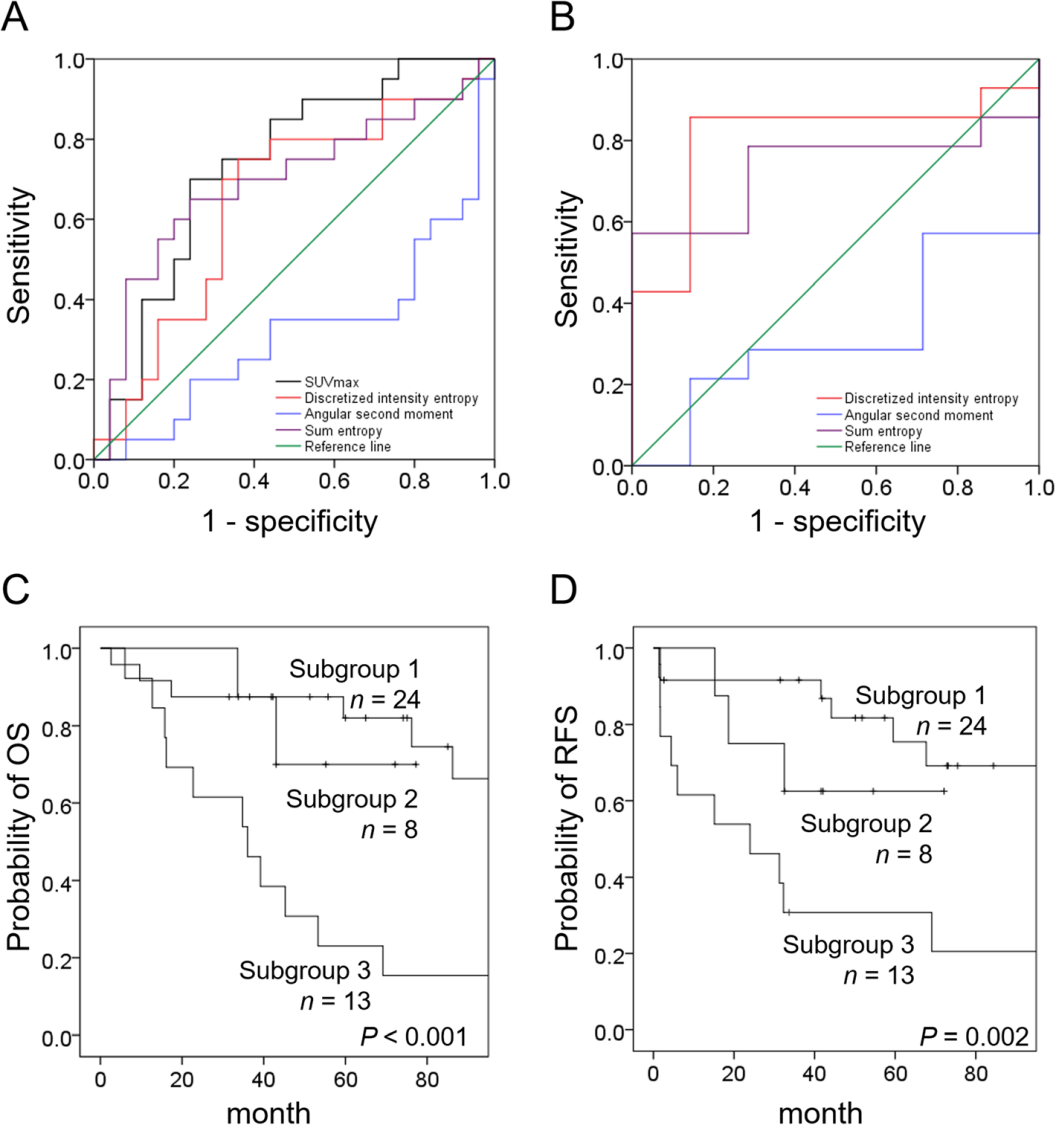
Kaplan-Meier plots of OS in relation to different PET parameters in the training group (**a**) and in the subgroup of patients with high SUV_max_ (**b**). Kaplan-Meier plots of OS (**c**) and RFS (**d**) according to the three PET risk patterns

As demonstrated in Fig. 1 and Supplementary Table 2, the results of ROC curve analysis in the training group revealed a significant association between tumor SUV_max_ and OS (C-index: 0.74, *P* = 0.007 with cutoff value of 6.67). Neither other PET tumor nor lymph node parameter was found to be related to RFS. Subgroup analysis revealed that in the training group, tumor discretized intensity entropy was significantly associated with OS in the subgroup of patients with SUV_max_ > 6.67 (*n* = 21; C-index: 0.81, *P* = 0.025; cutoff: 2.50). In contrast, none of the other tumor nor lymph node parameters was related to OS in the subgroup of patients with low SUV_max_ (*n* = 24).

Based on these findings, we devised the following PET prognostic system for predicting OS: patients in subgroup 1 (*n* = 24) had a low SUV_max_; patients in subgroup 2 (*n* = 8) had high SUV_max_ but low discretized intensity entropy; and patients in subgroup 3 (*n* = 13) had both high SUV_max_, and discretized intensity entropy. Patients in subgroup 3 showed the least favorable survival figures in terms of both OS and RFS (Fig. 1) and were therefore considered as having a high-risk PET pattern. The selection process of PET radiomics was demonstrated in Supplementary Figure 1. We also found a statistically significant inverse association between the presence of ACC and the high-risk PET pattern (ρ = − 0.41, *P* = 0.005).

### Development and validation of the prognostic model

**Fig. 2:**
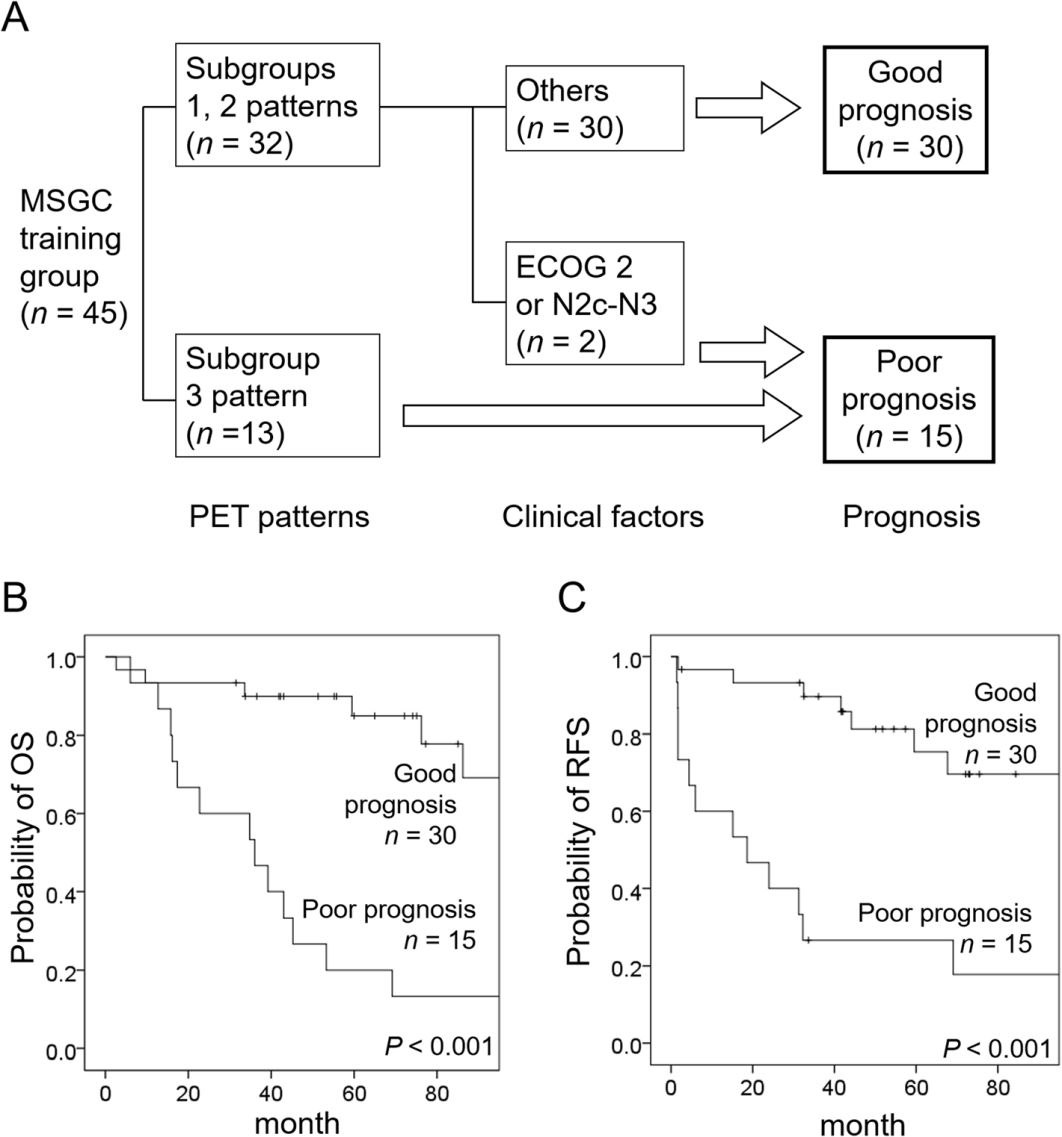
Development of the prognostic model based on the high-risk PET pattern and N2c-N3 stage or poor performance status (**a**). Kaplan-Meier plots of OS (**b**) and RFS (**c**) according to the prognostic model in the training group

**Fig. 3:**
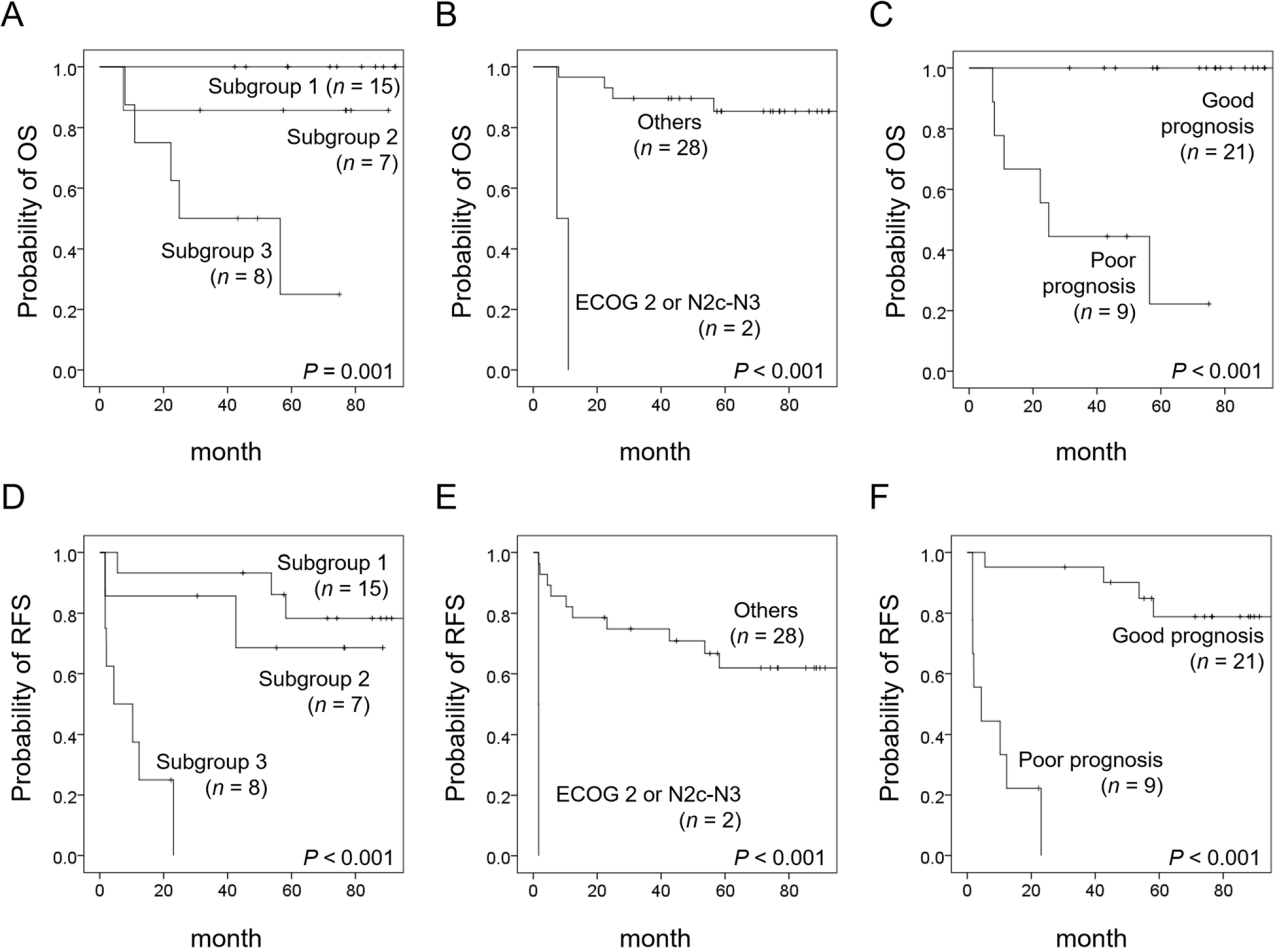
Kaplan-Meier plots of different survival endpoints according to PET risk patterns (**a**, overall survival; **d**, relapse-free survival), N2c-N3 stage or poor performance status (**b**, overall survival; **e**, relapse-free survival), and prognostic model (**c**, overall survival; **f**, relapse-free survival) in the validation group

All variables satisfied the proportional hazards assumption in Schoenfeld residuals tests. Bootstrap multivariate analysis based on 1,000 replications confirmed the significance of high-risk PET pattern and ECOG 2 or N2c-N3 status for both OS and RFS, Table 2. There was no significant correlation between different parameters in multivariate analyses of the training cohort. Therefore, these two variables were used to devise a prognostic model—which was developed by means of RPA and fivefold cross-validations. Interestingly, patients with the high-risk PET pattern had adverse outcomes, whereas those in subgroups 1 and 2 could be further stratified according to the ECOG2 or N2c-N3 status (Fig. 2). Finally, a total of 15 patients were classified as having a poor prognosis according to the prognostic model. We attempted to replicate these results in the validation group (*n* = 30). Figure 3 shows that patients with the high-risk PET pattern (i.e., high SUV_max_ and high discretized intensity entropy) and a positive ECOG 2 or N2c-N3 status had less favorable survival outcomes (both in terms of OS and RFS). The prognostic model effectively predicted both OS and RFS. This prognostic model worked effectively in predicting OS and RFS regardless of the histology type (all *P* < 0.001 in both the ACC and non-ACC subgroups). Because of the limited sample size (*n* = 30) and the presence of collinearity between the high-risk PET pattern and both AJCC stage and WHO high-risk histology (*ρ* = 0.480 and 0.498, *P* = 0.007 and 0.005, respectively), multivariable Cox proportional hazards regression analysis was not performed in the validation group.

**Table 2:** Multivariable Cox proportional hazards regression analysis of overall and relapse-free survivals in the training group

Variable	Overall survival		Relapse-free survival	
	HR (95% CI)	*P*	HR (95% CI)	*P*
Age (year)	0.69 (0.24-1.99)	0.580	2.47 (0.75-8.16)	0.207
WHO high-risk histology	0.56 (0.14-2.28)	0.545	0.70 (0.13-3.86)	0.639
Surgery versus non-surgery	2.00 (0.56-7.19)	0.444	0.29 (0.08-1.00)	0.107
AJCC stage III − IV versus I − II	2.43 (0.54-11.00)	0.366	3.10 (0.59-16.36)	0.239
ECOG 2 or N2c − N3	5.90 (1.54-22.60)	0.026	14.93 (2.38-93.72)	0.014
Subgroup 3 PET pattern	6.30 (2.14-18.56)	0.004	3.94 (1.44-10.76)	0.024

Abbreviations: *HR* hazard ratio; *CI* confidence interval; *WHO* World Health Organization; *AJCC* American Joint Committee on Cancer; *ECOG* Eastern Cooperative Oncology Group; *PET*

positron emission tomography

Results of performance of clinical parameters, high-risk PET pattern and the prognostic system in the entire study cohort (*n* = 75) were demonstrated in Supplementary Table 3. PET radiomics-based model (i.e., subgroup 3 PET pattern) had sensitivity of 63.0%, accuracy of 81.3% with C-index of 0.77 for OS. After integrated with ECOG 2 or N2c-N3 status, sensitivity, accuracy, and C-index could be improved to 74.1%, 85.3%, and 0.83, respectively. Similar findings were noted for RFS. Although those improvements in sensitivity and accuracy did not achieve significant level (McNemar test, *P* = 0.250), there was marginally significant in C-index improvement (*P* = 0.085 and 0.0918 for OS and RFS, respectively).

**Fig. 4:**
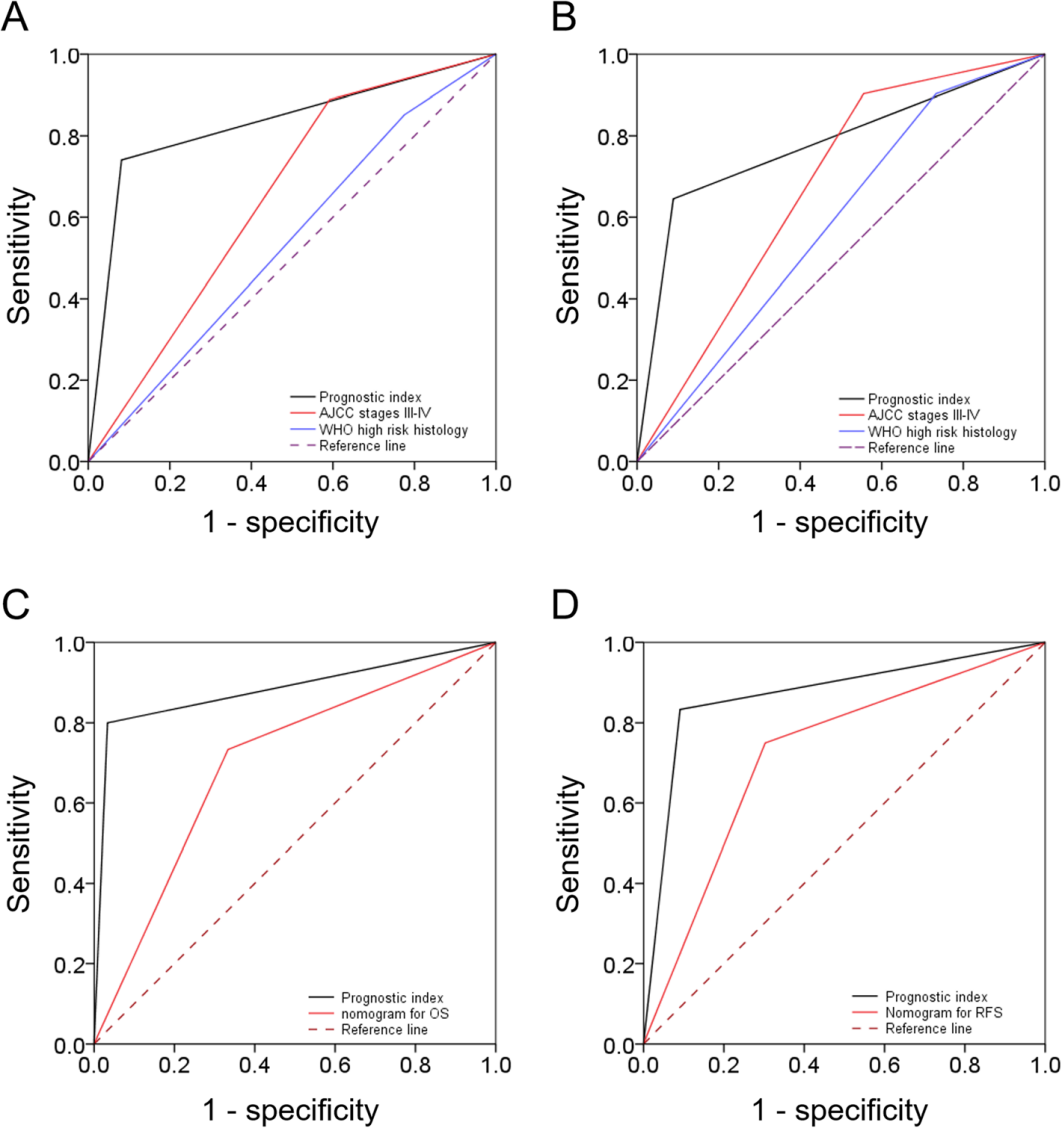
Receiver operating characteristic (ROC) curves showing that the C-index of the prognostic model was higher than those of AJCC stage III − IV and WHO high-risk histology for both OS (**a**) and RFS (**b**). For surgically treated patients (*n* = 45), the C-index of the prognostic model was higher than the nomogram for OS (**c**) and that for RFS (**d**)

The application of the prognostic system (*n* = 75) led to the identification of 24 (32.0%) patients with a poor prognosis. Importantly, the ability of the prognostic index to predict OS (C-index: 0.83) was significantly higher than those of the AJCC stage (C-index: 0.65, *P* = 0.005) and high-risk histology (C-index: 0.54, *P* < 0.001). Similar results were observed for RFS, with a higher C-index being evident for our prognostic model (0.78) compared with other variables (AJCC stage and high-risk histology, C-index: 0.68 and 0.59, *P* = 0.099 and 0.004, respectively; Fig. 4). Patients with a poor prognosis were more likely to have disease recurrences (odds ratio: 18.18; 95% confidence interval: 5.14 − 64.36, *P* < 0.001). Three cases with tumor volumes < 64 voxels had lower SUV_max_ (3.89, 4.55, 5.47) and were not classified as high-risk PET patterns. All had survived during the study period without relapse. Remove those three lesions had no impacts on subsequent analyses.

Our prognostic model was further compared with two previously published nomograms for predicting OS [13] and RFS [14]. Although the two nomograms effectively predicted survival endpoints in our sample (C-index for OS: 0.70, *P* = 0.007; C-index for RFS: 0.72, *P* = 0.004), the current prognostic model showed superior performance for OS (C-index: 0.88 vs. 0.70, *P* = 0.017) and for RFS (C-index: 0.87 vs. 0.72, *P* = 0.004).

## Discussion

This extended study enrolled MSGC of all kinds of histology. More radiomic data were investigated using the testing and validation method. Nevertheless, the PET parameters we found were identical to our previous paper. The results indicate that tumor SUV_max_, discretized intensity entropy, and ECOG 2 or N2c-N3 status are independently associated with survival endpoints in patients with MSGC. These variables were used to devise and validate a prognostic model that was clinically useful for improving outcome stratification in this patient group. Notably, our prognostic model was found to have a higher predictive power as compared with AJCC staging, WHO classification, and currently available nomograms. Patients identified as having a poor prognosis according to our model are unlikely to benefit from current treatment modalities and are ideal candidates for novel treatment approaches in clinical trials.

To our knowledge, this study is the first to specifically focus on the prognostic significance of PET radiomics in MSGC. Owing to the robustness and high reliability of SUV_max_ and discretized intensity entropy obtained from different PET imaging systems [25, 26], these parameters have the potential to serve as image biomarkers in multicenter clinical trials. In our study, tumor SUV_max_ was identified as an independent prognostic factor in patients with MSGC. This finding may be explained by the positive correlation between SUV_max_ values and the extent of glucose transporter type-1 (GLUT-1) expression in tumor tissue [31]. An increased expression of GLUT-1 reflects a high biological aggressiveness of salivary gland malignancies and has been associated with poor clinical outcomes [32, 33]. Conversely, ACC—which generally shows a better OS compared with other histology types—is characterized by a reduced GLUT-1 expression [34] paralleled by lower SUV_max_ values. Genomic and phenotypic tumor heterogeneity may influence both response to therapy and clinical outcomes [35, 36]. In this scenario, discretized intensity entropy—which can reflect the spatial heterogeneity of GLUT-1 expression—has been shown to predict survival in patients with head and neck cancer [37] and pancreatic cancer [38]. Discretized intensity entropy can also be a reflection of the tumor microenvironment (e.g., vascularization, cell density, biological aggressiveness, and hypoxia) [39, 40], which may in turn influence disease recurrences in MSGC. The extent to which PET heterogeneity does actually reflect biological heterogeneity at cellular, molecular, and genetic levels has not been completely understood. For example, PET heterogeneity indices do not show significant associations with the mutation burden and genetic heterogeneity in lung cancer [41]. It is therefore possible that such PET parameters may actually have an independent prognostic significance.

Recent studies have shown that the number of positive lymph nodes—but not lymph node size, extranodal extension, and lower neck involvement—can predict survival outcomes in SGC [42, 43]. Because an N2c-N3 status likely reflects a higher number of nodal metastases, the adverse prognostic impact of this variable as observed in our study is not surprising. The unfavorable prognostic significance of poor PS is also in line with a previous study conducted in SGC [44]. Interestingly, we were also able to confirm the utility of two previously described nomograms (originally developed for major salivary gland tumors) in the prediction of clinical outcomes in MSGC.

Although radiomics-based clinical-decision-support systems (CDDS) are evolving rapidly, significant challenges still remain (including an unambiguous data collection and an accurate integration of different features) [19]. In keeping with the principles summarized in the radiomics quality score, the present study was designed to (1) validate the findings originally obtained in a testing group, (2) include multivariable analysis in the statistical methodology, and (3) compare the predictive ability of our model with that of currently recognized prognostic factors. Moreover, all of the PET images were acquired using standard imaging protocols and the cutoff values for SUV_max_ and discretized intensity entropy were identical to those reported in a previous study [17]. Notably, the application of our model led to the identification of a subgroup of MSGC patients that is unlikely to benefit from current therapies. Despite these strengths, our findings should also be interpreted in the context of some limitations. First, the retrospective, single-center nature of our extended study is prone to bias and limits the generalizability of our conclusions. Consequently, our findings need to be confirmed and expanded in larger independent, multicenter investigations. However, the rarity of MSGC still severely hampered large scale radiomics investigations. Second, we enrolled three cases (4%) with smaller tumors (< 64 voxels). Although there was no influence on subsequent analysis, the texture indices may provide more valuable complementary information for tumors with larger volumes [45]. Third, two PET systems were involved in this study. Although SUV_max_ and discretized intensity entropy were insensitive to device in our study, the variance of voxel size may considerably influence the agreement of measurements [20, 46]. Therefore, harmonizing voxel sizes may have the potential to remove the device-effect although more studies are needed to state its effects with certainty.

## Conclusions

We devised a novel prognostic model that incorporates [^18^F]FDG PET/CT radiomics and may help refine outcome prediction in patients with MSGC.

## Data Availability

The datasets used and/or analyzed during the current study are available from the corresponding author on reasonable request.
